# Simultaneous care in oncology: Assessment of benefit in relation to symptoms, sex, and age in 753 patients

**DOI:** 10.3389/fonc.2022.989713

**Published:** 2022-10-14

**Authors:** Antonella Galiano, Stefania Schiavon, Mariateresa Nardi, Irene Guglieri, Ardi Pambuku, Rosalba Martino, Maital Bolshinsky, Sabina Murgioni, Rossana Intini, Caterina Soldà, Dario Marino, Francesca Daniel, Chiara De Toni, Chiara Pittarello, Benedetta Chiusole, Alessandra Anna Prete, Davide Bimbatti, Floriana Nappo, Mario Caccese, Francesca Bergamo, Antonella Brunello, Sara Lonardi, Vittorina Zagonel

**Affiliations:** ^1^ Department of Oncology, Oncology Unit 1, Veneto Institute of Oncology IOV-IRCCS, Padua, Italy; ^2^ Pain Therapy and Palliative Care Unit, Veneto Institute of Oncology IOV-IRCCS, Padua, Italy; ^3^ Clinical Nutrition Unit, Veneto Institute of Oncology IOV-IRCCS, Padua, Italy; ^4^ Oncology Unit, “Villa Scassi” Hospital, Genua, Italy; ^5^ Hospital Psychology, Veneto Institute of Oncology IOV-IRCCS, Padua, Italy; ^6^ Department of Oncology, Oncology Unit 3, Veneto Institute of Oncology IOV-IRCCS, Padua, Italy

**Keywords:** simultaneous care, early palliative care (EPC), symptom assessment, advanced cancer, end of life chemotherapy, patient-centered care

## Abstract

**Background:**

Early activation of palliative care for patients with advanced cancer is central in the treatment trajectory. At the Veneto Institute of Oncology, a simultaneous-care outpatient clinic (SCOC) has been active since 2014, where patients are evaluated by an oncologist together with a palliative care team. Recently, we reported on consecutive patients admitted at SCOC from 2018 to 2021 in terms of appropriateness, process, and outcome indicators. Here, we report further analysis in the same group of 753 patients, evaluating other parameters and the correlation between symptom intensity, gender, age, and survival.

**Methods:**

SCOC data were retrieved from a prospectively maintained database.

**Results:**

Among the patients, 42.2% were women, and the median age was 68 years, with 46.7% of patients aged ≥70 years. The most prevalent disease type was gastrointestinal cancer (75.2%), and 90.9% of the patients had metastatic disease. The median score for the distress thermometer was 4; the vast majority of the patients (98.6%) reported physical problems, and 69.4% presented emotional issues. Younger women demonstrated a significantly greater median distress than other patients (p=0.0018). Almost all symptoms had a higher prevalence on the 0–3 Edmonton Symptom Assessment Scale (ESAS) score, except for fatigue. About 43.8% of the patients received systemic anticancer treatment (SAT) in the last 60 days of life, 15.0% of whom received SAT in the last month and 3.1% in the last 2 weeks. For some symptoms, women frequently had more ESAS >3. Pain and nausea were significantly less reported by older patients compared with younger adults. Men had a lower risk of having MUST score ≥ 2 (p=0.0311). Men and older patients showed a lower prognosis awareness (p=0.0011 and p=0.0049, respectively). Older patients received less SAT within the last 30 days of life (p=0.0006) and had death risk decreased by 20.0%.

**Conclusion:**

Our study identified two subgroups of patients with advanced cancer who require special attention and support due to important symptoms’ burden detected by Patient Reported Outcome Measures tests: women and younger adults. These categories of patients require special attention and should be provided early access at SCOC. The role of an oncologist remains crucial to intercept all patients in need of early palliative care and balancing trade-offs of anticancer treatment in advanced metastatic disease.

## Introduction

There is mounting evidence on the crucial role of early activation of palliative care in patients’ cancer journey, especially in the advanced stage of the disease (1). Indeed, numerous studies have shown that this approach improves the quality of life and, in some cases, even patients’ survival (2–6). As a result, early palliative care is now recommended by most prominent international oncology scientific societies and is included in their guidelines (7–10).

Despite such evidence, outcomes obtained with this approach are not consistently reported and appear to be related to several key elements through which early palliative care benefits patients and caregivers (11). In addition, the heterogeneity related to different organizational models, the availability of palliative care teams, as well as the cultural and social-health aspects across different countries, to date, do not allow suggesting a unique model for early palliative care delivery (11, 12).

The Veneto Institute of Oncology (IOV) takes charge of more than 5,000 new cancer patients in need of systemic treatment per year. IOV is an Organization of European Cancer Institutes (OECI) certified Comprehensive Cancer Center, and since 2012, the Oncology Department has obtained the European Society of Medical Oncology (ESMO) certification as a Designated Center of integration of oncology and palliative care (ESMO-DC). Since 2017, the Institute has adopted a procedure with standardized referral criteria through (1) routine screening of supportive care needs at oncology visits; (2) filling in a referral form by oncologists at the time of the visit; the form was defined by oncology and palliative care teams for identifying patients with palliative care needs; (3) a system in place to trigger referral when patients meet the criteria; and (4) activation of simultaneous care outpatients clinic (SCOC), in which the oncologist and the palliative care team (a palliative care physician, a physician specialized in clinical nutrition, a psycho-oncologist, and a nurse navigator) assess together, through validated tools, the needs of patients with the aim to deliver personalized, timely patient-centered care and improve patient and caregiver outcomes (13). This embedded model meets internationally agreed criteria for optimizing the early inclusion of palliative care in the care pathway (14, 15). In order to ensure an early referral of patients with metastatic disease, patients’ assessment is based on symptom’s burden and life expectancy, and through the activation of a simultaneous care clinic, the oncologist and the palliative care team share the patient’s journey (13). Recently, we reported the data on our series of 753 patients evaluated at SCOC from January 2018 to December 2021 in terms of indicators of appropriateness, process, and outcome provided by the Institute's procedure (13). In this work, we report the data from further analyses performed in the same group of 753 patients, evaluating a number of other parameters and the correlation between symptom intensity, gender, age, and survival.

## Patients and methods

### Patients

This study was conducted at the Veneto Institute Oncology (IOV), Padua, Italy. The study population was composed of patients referred to SCOC between January 2018 and December 2021. Selection criteria were the availability of the referral form filled in by the oncologist and cancer-directed treatment planned. SCOC data were retrieved from a prospectively maintained database: demographic and clinic information, distress thermometer (DT), Edmonton Symptom Assessment Scale (ESAS), and Malnutrition Universal Screening Tool (MUST). These three scales (DT, ESAS, and MUST) are used because of the following characteristics:

- The DT is a simple tool developed by the National Comprehensive Cancer Network (NCCN), which provides effective screening for symptoms of distress. The instrument is a self-reported tool using a Likert rating scale (0 to 10) and additionally identifies sources of distress using a Problem List (PL) (16).
- The ESAS is a measure of symptom burden that includes a Likert rating of nine symptoms, on a scale from 0 (best) to 10 (worst), which has been adopted for routine needs screening during the SCOC visit (17).
- The MUST identifies patients who are malnourished or are at risk of malnutrition; a score of 0 indicates a low risk of malnutrition, a score of 1 indicates medium risk, and a score ≥ 2 indicates a high risk (18, 19).

We also analyzed whether there were significant differences by gender, age (age less than, or equal to, and over 70 years), and type of cancer, with regard to a series of variables:

1. DT

2. ESAS: type of symptoms and intensity

3. MUST

4. Awareness of the cancer prognosis (total, partial, absent)

5. Systemic anticancer treatment (SAT) at the end of life (last 60, 30, and 14 days)

6. Unplanned visits to the emergency room (ER)

7. Place of death (hospital *vs*. hospice or home)

8. Actual survival at the time of SCOC referral

### Statistical analysis

The patients’ characteristics were described by descriptive analysis. The comparisons were tested using chi-square tests, Fisher’ exact tests, and log-rank tests, as appropriate. For the survival analysis, all patients entered into the study at the date of SCOC were followed up until 31 January 2022 or the date of death, whichever came first. Median survival was calculated with the Kaplan–Meier method. The following variables were analyzed: ESAS, MUST, territorial services activation, prognosis awareness, chemotherapy within the last 30 days of life, unplanned access in the ER, place of death, actual and estimated survival; figures were drawn for gender and age comparisons including only the significant results. The place of death and the end-of-life chemotherapy were assessed for deceased patients. Cox’s proportional hazards model was fitted to the data to evaluate the association between the actual survival and the variables of interest (gender, age class, and tumor site). Logistic, multinomial, and cumulative logit models were used to test the association between the category variables, previously considered in the bivariate analysis, and the variables of interest. R Version 4.2.0 was used to perform all statistical analyses. The level of significance was set at 5%.

## Results

Demographic and patients’ clinical characteristics are shown in **Table 1**. Among the patients, 318 were women (42.2%), and the median age was 68 years (range: 60–76 years), with 352 patients (46.7%) aged 70 years and older. The most prevalent disease type was gastrointestinal cancer (566 patients, 75.2%). A total of 661 (87.8%) patients had a Karnofsky performance status (KPS) ≥70, 684 (90.9%) patients had metastatic disease, and 223 (29.6%) patients received more than two lines of therapy. The time from cancer diagnosis to the first SCOC visit was less than 1 year for 351 patients (51.8%). The median survival of the overall population from SCOC visit was 7.3 months (95% CI: 6.5–8.0).

**Table 1:** Patients’ characteristics.

Characteristics	Patients (n)753	(%)100
**Gender**:		
Men	435	(57.8)
Women	318	(42.2)
**Age at referral (years)**:		
Median (IQR)	68	(60–76)
< 70 years	401	(53.3)
≥ 70 years	352	(46.7)
**Tumor site**:		
Gastrointestinal (GI)	566	(75.2)
Genitourinary (GU)	113	(15.0)
Other (sarcoma, lymphoma, gynecological)	74	(9.8)
**Karnofsky Performance Status**:		
≥70	661	(87.8)
50-60	92	(12.2)
**Tumor stage**:		
Locally advanced	47	(6.2)
Metastatic	684	(90.9)
Missing	22	(2.9)
**Treatment line**:		
First line	338	(44.9)
Second line	192	(25.5)
Third or further lines	223	(29.6)
**Years since cancer diagnosis**:		
≤1	351	(51.8)
>1	326	(48.2)
**Survival from the SCOC visit (months)**:		
Median (95% CI)	7.3	(6.5-8.0)

### Symptom’s burden

**Figure 1:**
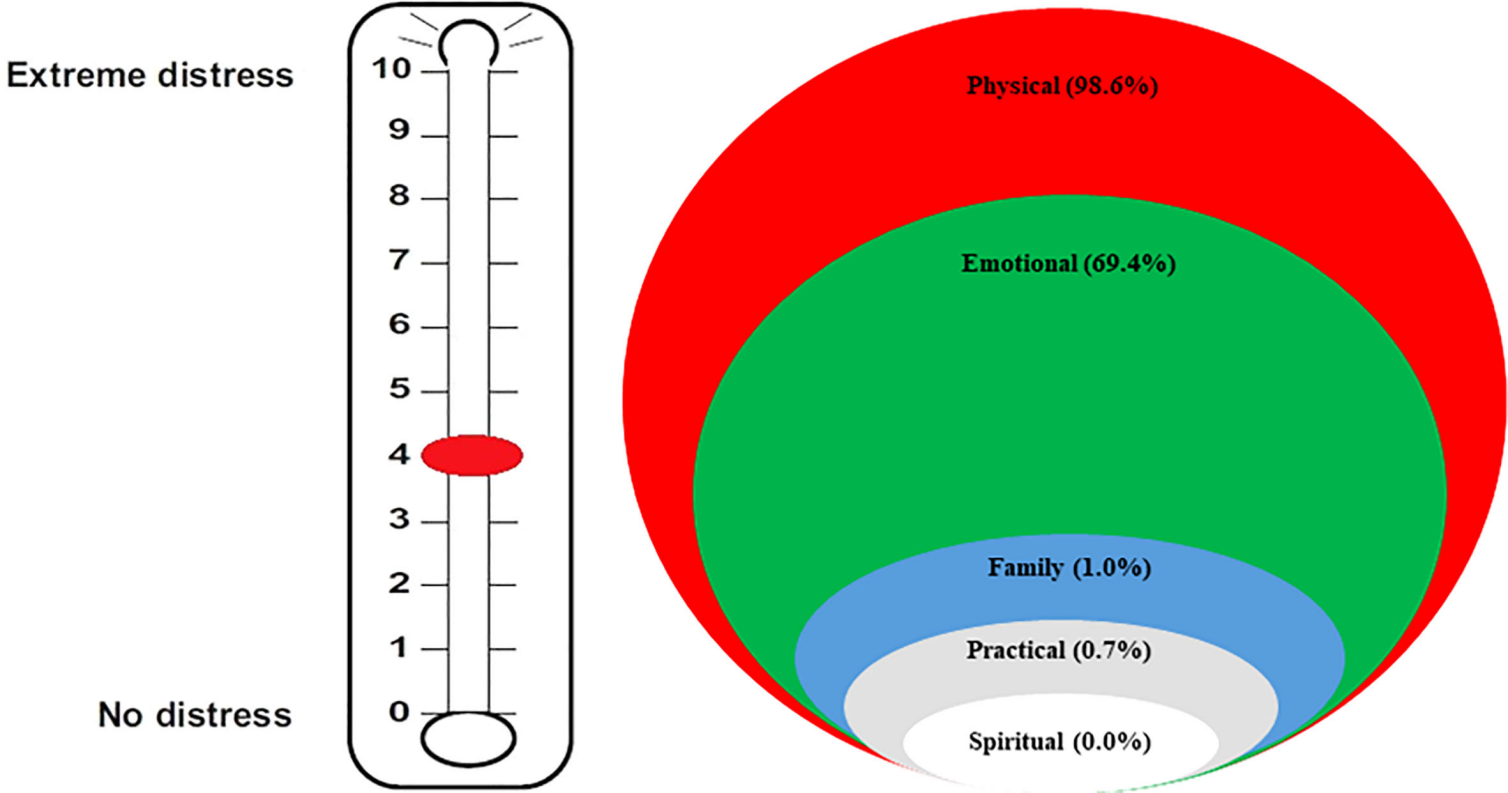
Results according to distress thermometers: median score and burden of distress in the different areas.

**Figure 2:**
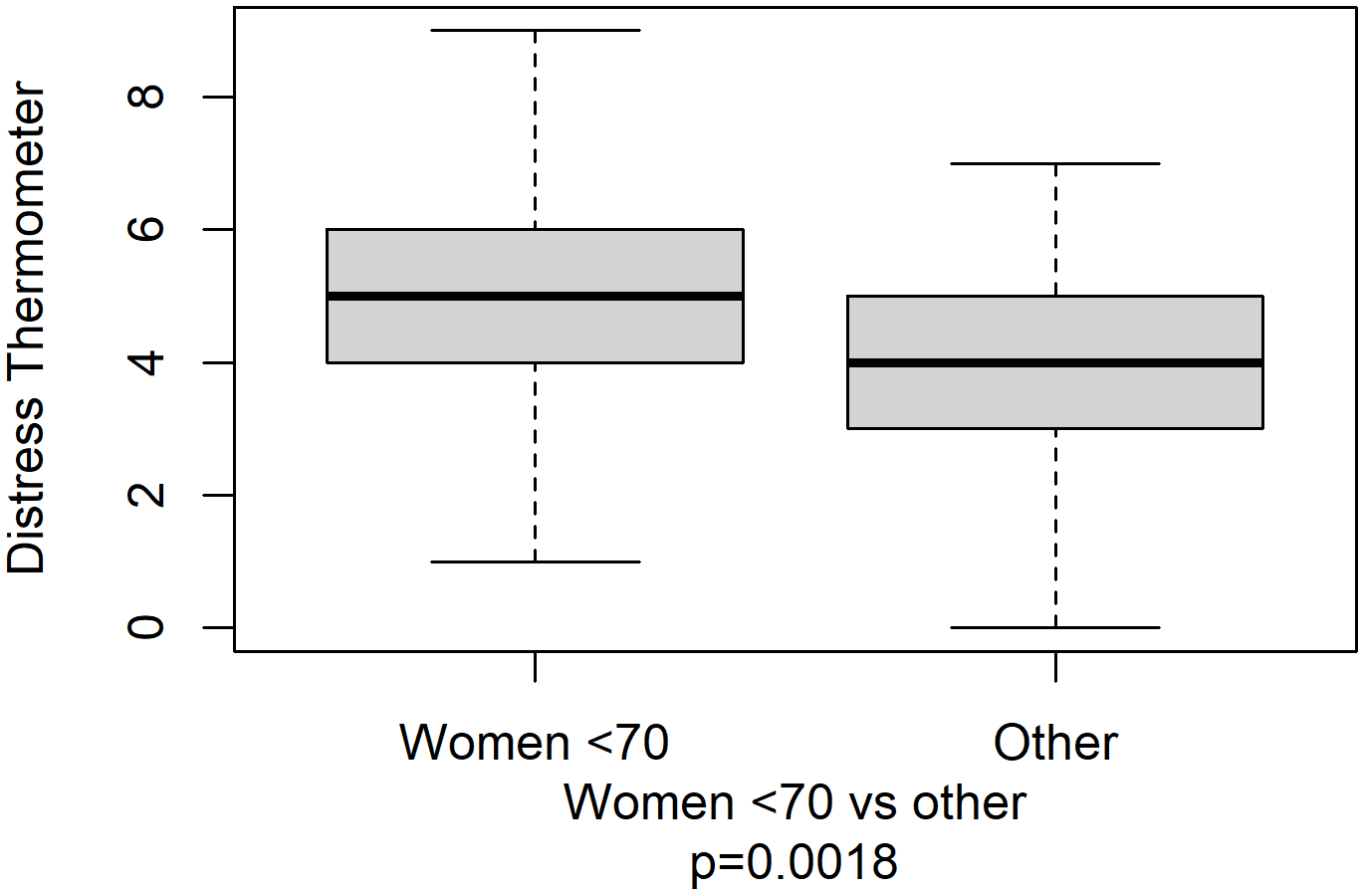
Distress thermometer’s boxplot by women under 70 years versus others.

The median score for DT was 4 (range: 0–9), with the vast majority of patients (98.6%) reporting physical problems and more than half (69.4%) presenting emotional issues, as shown in **Figure 1**. Family and practical problems and spiritual concerns were present in a small percentage of patients (1.0%, 0.7%, and 0.0%, respectively). Younger women reported a significantly greater median distress compared with other patients (5 *vs*. 4, p=0.0018, **Figure 2**).

**Figure 3:**
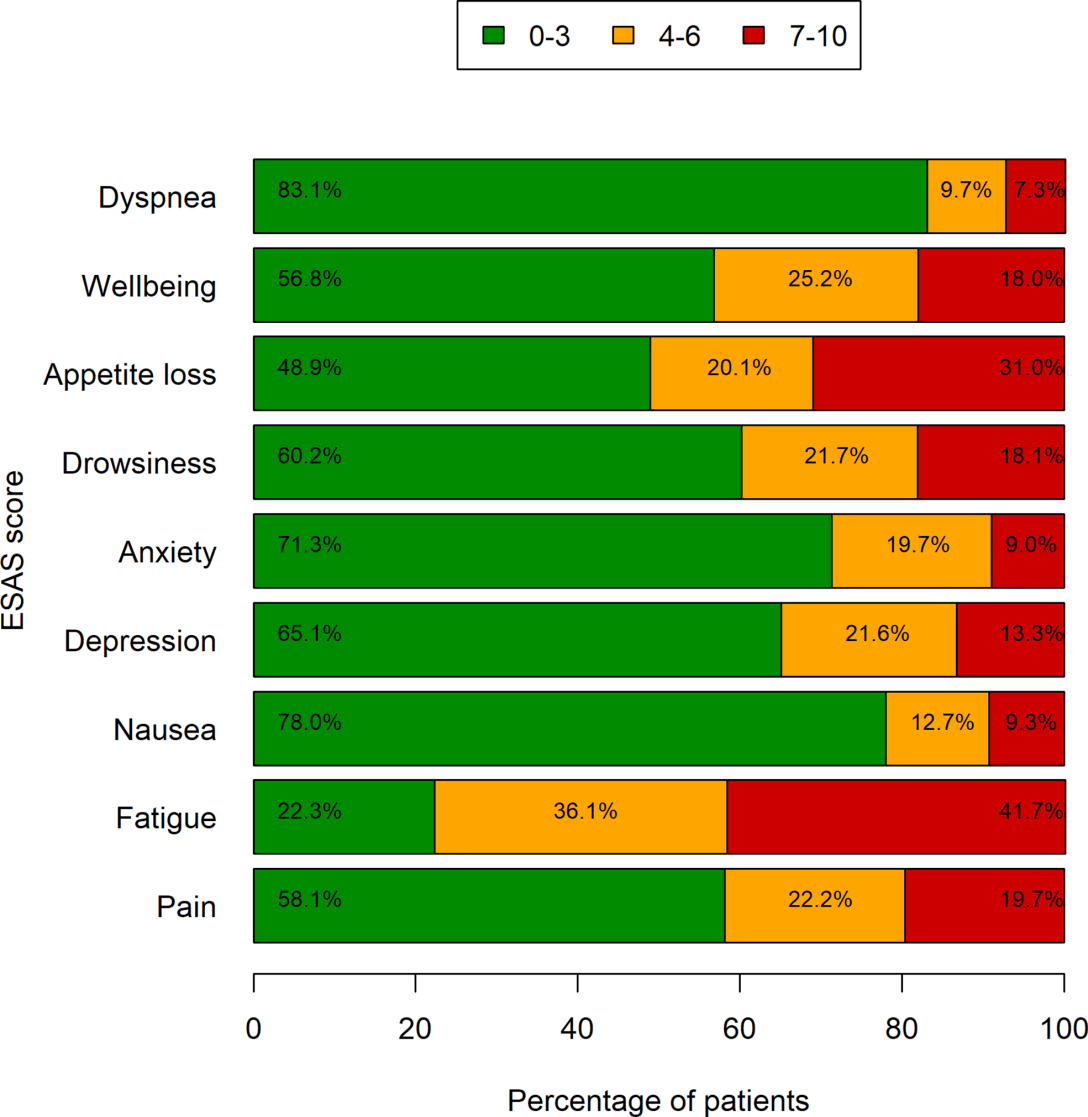
Symptom severity by ESAS. Bars represent the frequencies of symptoms grouped by three levels of severity (data missing in up to 92 patients).

ESAS symptoms by three levels of severity are shown in **Figure 3**. Almost all symptoms had a higher prevalence in the 0–3 score range, except for fatigue, which was experienced with an intensity of 7–10 in 281 patients (41.7%). A total of 175 patients had three or more symptoms with an intensity of 7–10. The median survival for these patients was 5.6 months (95% CI: 4.7–7.4), whereas the median survival for the other patients was 7.7 months (95% CI: 6.8–8.6; log-rank test’s p-value=0.0232) (**Table 2**).

**Table 2:** Survival according to worse symptom’s burden by ESAS.

≥ 3 ESAS, score 7–10	Patients (%)	Events	Median survival (months)	95% CI	p-value
**Yes**	175 (23.2)	128	5.6	[4.7–7.4]	0.0232
**No**	578 (76.8)	423	7.7	[6.8–8.6]	

### Systemic anticancer treatment at the end of life

As of 31 January 2022, 552 (73.3%) patients were deceased. The median number of days between the last administration of SAT and patient death was 66 (range: 1–1193 days). A total of 242 patients (43.8%) received SAT in the last 60 days of life, among which 83 (15.0%) received SAT during the last month and 17 (3.1%) in the last 2 weeks of life (**Table 3**). The median age of patients who received SAT in the last 30 days was 63 years (IQR: 57–69), which is lower than the rest of the group (68 years, IQR: 60–76, p=0.0005). Nearly half (47.0%) of the patients who received SAT in the last 30 days of life were being treated in the first line. The median survival from the SCOC visit for these patients was 3.4 months (95% CI: 1.8–4.5) compared with 5.9 months (95% CI: 5.5–6.4) for the other patients (p <0.0001). For this group of patients, the hospital was the more frequent place of death (60.0% *vs*. 25.5% in other patients, p<0.0001). There were no differences with regard to unplanned access to the ER, hospital admission, number of lines of treatment, and years for patients treated or not treated with SAT in the last month of life.

**Table 3:** Systemic anticancer treatment (SAT) at the end of life.

SAT	Last 60 days n (%)	Last 30 days n (%)	Last 14 days n (%)	Median survival(months)	95% CI	p-value
**Yes**	242 (43.8)	83 (15.0)	17 (3.1)	3.4	[1.8–4.5]	<0.0001
**No**	310 (56.2)	469 (85.0)	535 (96.9)	5.9	[5.5–6.4]	

### Results by gender

**Figure 4:**
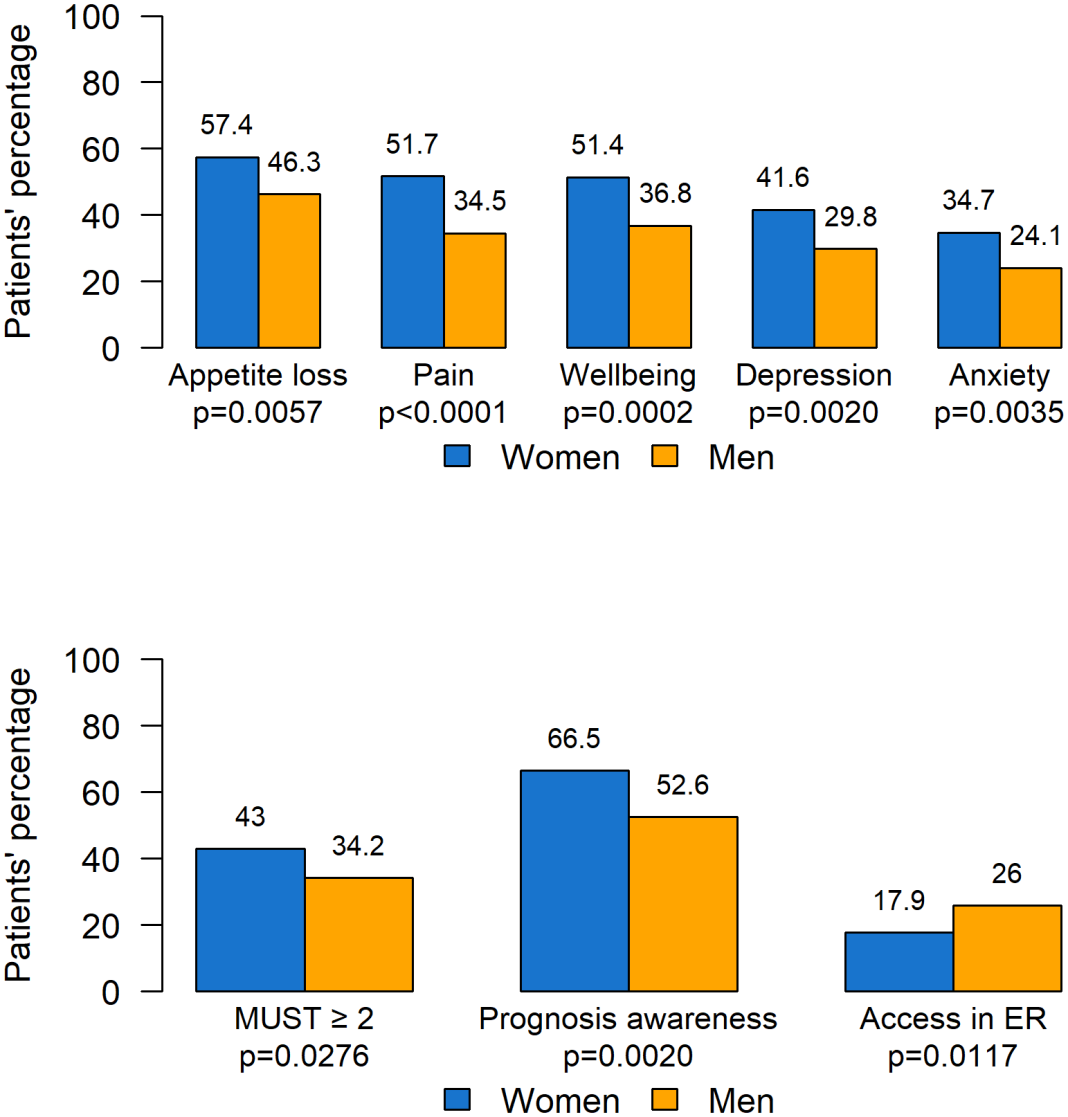
Statistically significant difference according to gender. ER, Emergency room.

ESAS symptoms with intensity greater than 3 were differently distributed according to gender, with women reporting higher prevalence of appetite loss, pain, wellbeing, depression, and anxiety compared with men (see **Figure 4**). No difference was observed for dyspnea, which was the only symptom more frequent in men (women: 14.8% *vs*. men: 18.6%, p=0.2388, data not shown).

MUST scores were worse for women, with 117 (43.0%) having a score ≥ 2 (patients at higher risk of malnutrition), compared with men (130, 34.2%, p=0.0276).

More women were found to have complete awareness of the disease prognosis compared to men (66.5% *vs*. 52.6%). Moreover, men presented more unplanned access to the ER (26.0% *vs*. 17.9% for women, p=0.0117). No significant differences according to gender were found in territorial services activation, the number of patients undergoing SAT in the last 30 days of life, the place of death, and survival.

### Results by age

**Figure 5:**
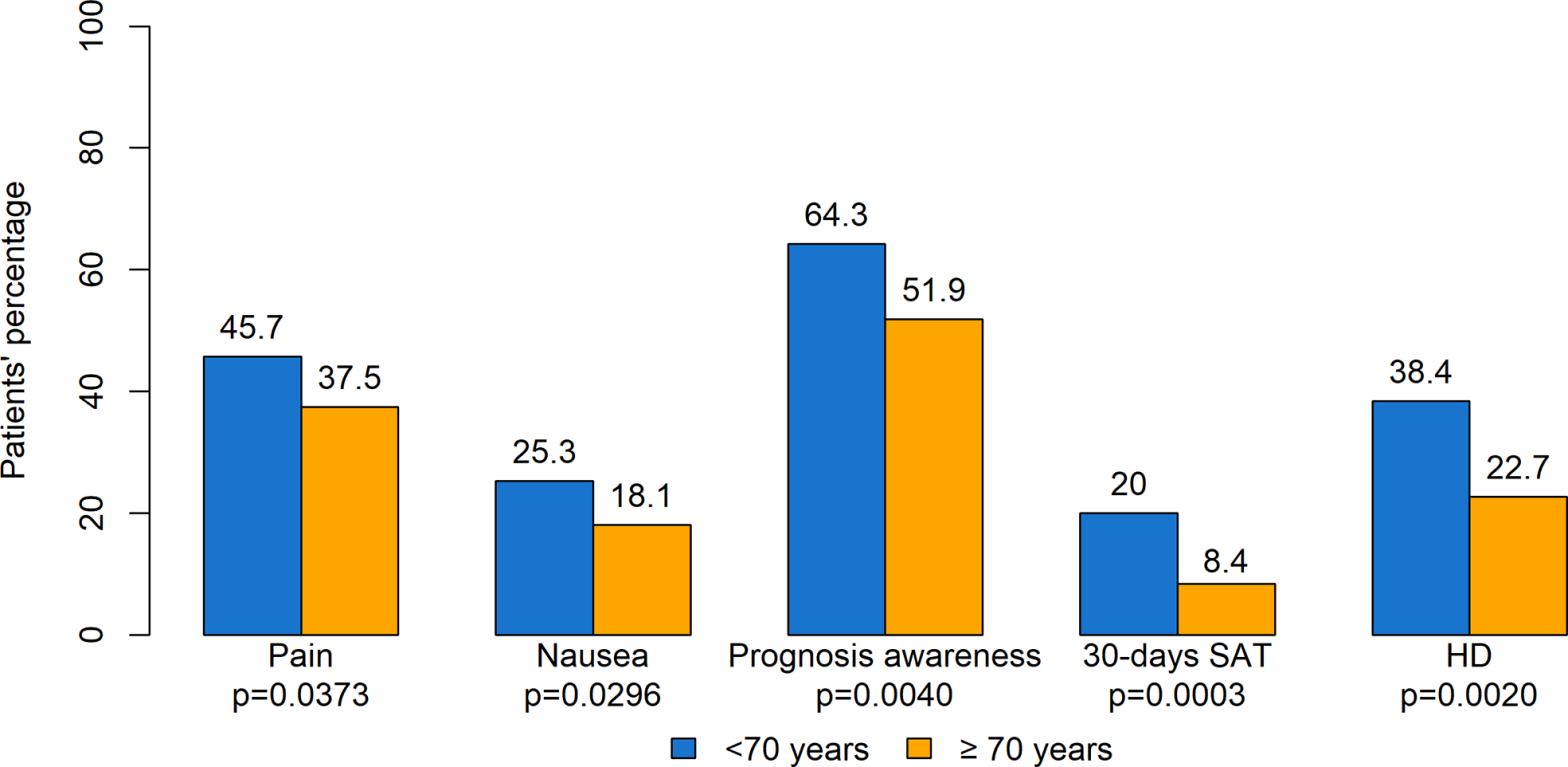
Statistically significant difference according to age. SAT, systemic anticancer therapy; HD, hospital death.

**Figure 5** summarizes significant differences according to the age of patients. With regard to ESAS, pain (p=0.0373) and nausea (p=0.0296) had a higher prevalence in younger compared to older patients. As for prognosis awareness, 209 patients (64.3%) aged <70 years reported total prognosis awareness compared with 148 (51.9%) patients aged ≥ 70 years. SAT in the last 30 days of life was administered to 63 (20.0%) adults aged <70 years and to 20 (8.4%) older patients (p=0.0003). Also, a significant difference was observed with regard to the place of death, occurring in hospital for 38.4% younger subjects compared with 22.7% for older patients. Moreover, older patients had better survival, with median survival for adults aged <70 years being 6.3 months (95% CI: 5.7–7.2) compared with 8.8 months (95% CI: 7.5–10.1) for patients aged ≥ 70 years (p=0.0006). No other significant differences were found.

### Multivariate analysis

Regression models were developed taking into account the variables of interest. As reported in **Table 4**, multivariate analysis confirmed the statistically significant difference in the ESAS score by gender (women’s ESAS score >3 for the symptoms pain, nausea, depression, anxiety, appetite loss, and wellbeing), MUST (lower risk of having a score ≥ 2 in men), and a higher awareness of cancer prognosis in women. Patients aged 70 years and older also had 20% lower risk of death (p=0.0072). Elderly patients received less SAT within the last 30 days of life as well as in the last 2 months (OR=0.6, p=0.0058, data not shown). With regard to the tumor site, only mortality risk resulted significant in multivariate analysis, being 1.5 times higher for GI cancers compared with other cancer types (p=0.0099).

**Table 4:** Results by multivariate analysis.

		OR	95% CI	p-value
**ESAS (ref: score ≤ 3)**				
**- Pain**				
Gender (ref: Women)	Men	0.5	(0.3–0.6)	<0.0001
Age class (ref: < 70 years)	≥ 70	0.7	(0.5–0.9)	0.0149
Tumor site (ref: Other)	GU	2.3	(1.2–4.6)	0.0155
**- Nausea**				
Gender (ref: Women)	Men	0.7	(0.4–1.0)	0.0276
Age class (ref: < 70 years)	≥ 70	0.6	(0.4–0.9)	0.0103
**- Depression**				
Gender (ref: Women)	Men	0.6	(0.4–0.8)	0.0008
**- Anxiety**				
Gender (ref: Women)	Men	0.6	(0.4–0.8)	0.0016
**- Appetite loss**				
Gender (ref: Women)	Men	0.6	(0.5–0.8)	0.0031
**- Wellbeing**				
Gender (ref: Women)	Men	0.5	(0.4–0.7)	<0.0001
Tumor site (ref: Other)	GU	2.4	(1.2–4.8)	0.0111
**- Dyspnea**				
Tumor site (ref: Other)	GI	0.5	(0.3–1.0)	0.0440
**MUST (ref: score 0–1)**				
Gender (ref: Women)	Men	0.7	(0.5–1.0)	0.0311
**PROGNOSIS AWARENESS (ref: Absent)**				
Gender (ref: Women)	Men	0.6	(0.4–0.8)	0.0011
Age class (ref: < 70 years)	≥ 70	0.6	(0.5–0.9)	0.0049
**SAT^ AT THE END OF LIFE (ref: > 30 days)**				
Age class (ref: < 70 years)	≥ 70	0.4	(0.2–0.7)	0.0006
UNPLANNED ER* VISITS (ref: No)				
Gender (ref: Women)	Men	1.6	(1.1–2.3)	0.0110
**PLACE OF DEATH (ref: Hospital)**				
Gender (ref: Women)	Men	0.6	(0.4–1.0)	0.0294
Age class (ref: < 70 years)	≥ 70	2.1	(1.3–3.3)	0.0028
**SURVIVAL**		**HR**		
Age class (ref: < 70 years)	≥ 70	0.8	(0.7–0.9)	0.0072
Tumor site (ref: Other)	GI	1.5	(1.1–2.0)	0.0099

^SAT, systemic anticancer therapy.

*ER, emergency room.

OR, odds ratio; HR, hazard ratio.

## Discussion

Early integration of palliative care in the cancer patient’s care path is today regarded as an essential goal to optimize the quality of life in the advanced stage of the disease and is best delivered in outpatient clinics (1). There is no single model of palliative care that is appropriate for all settings (11). The embedded model put in place in our Department, in which the palliative care team shares the SCOC with the oncologist, meets all the criteria proposed by international consensus to ensure timely activation of palliative care (20). This innovative organizational model allows intercepting cancer patients in an advanced stage of disease who need global care. The needs of each patient are addressed through the systematic use of validated Patient Reported Outcome Measures (PROMs), and this allows the customization of the patient’s journey and future end-of-life care decisions. In particular, the joint presence of an oncologist together with the palliative care team facilitates dialogue with patients and caregivers on advance care planning, end-of-life provisions, and preferential death location (21). Sharing resources between oncology and palliative care services is also cost-effective and may encourage collaborative education and research (11, 15). New organizational models are a challenge and an important resource to guarantee assistance to cancer patients also in the COVID era and in every phase of the illness trajectory (Andrè Ilbawi, WHO Cancer Control Officer, Opening Session at ASCO 2022 congress, the 4^th^ June 2022).

A previous paper by our group described the organizational model of embedded systemic early palliative care for patients with advanced cancer and the results of the last 4 years of activity as assessed through indicators of outcome, process, and appropriateness (13). The present work reports the results concerning symptoms burden, SAT at the end of life, difference by age, gender, and survival in the same group of 753 patients referred to SCOC in the period 2018–2021.

The *DT*, first described by Roth et al. in 1998, was developed for assessing distress in cancer patients (16). Since then, several experiences have been published regarding this easy-to-use tool with the ability to intercept at a glance the main problems of the patient (22, 23). A wide proportion of cancer patients, ranging between 25% and 60%, report distress when they are assessed (24). The median DT score in our patients was 4 (range: 0–9), with 98.6% of the patients experiencing physical problems, 69.4% emotional problems, and only 1.0%, 0.7%, and 0.0% reporting familiar, practical, and spiritual problems, respectively. This very low frequency of practical, family, and spiritual problems might look surprising, taking into account other studies, particularly American experiences, in which up to 80% of the patients with cancer attribute their distress to financial stressors (25, 26). This may be due to several reasons, including the different Italian healthcare system (which guarantees the coverage of most of cancer therapies compared with the American insurance system), the different social aspects of family relationships, and more widespread religious beliefs, as well as the patients’ reluctance to involve the doctor on problems other than oncological disease.

*Fatigue* was highly relevant in our patients’ population, reported by 92.7% with the DT and confirmed by the ESAS assessment. Notably, for ESAS > 3, fatigue was detected in 77.8% of the patients. Indeed, fatigue constitutes the most distressing patient-related symptom in terms of intensity and frequency that negatively affects their quality of life (27–29), although, unfortunately, nothing at this time has been shown to effectively relieve this symptom (30). Fatigue is multifactorial, related both to the treatment (surgery, chemotherapy, and radiation therapy) and to the tumor itself. It is usually underestimated by physicians, and its management remains one of the greatest unmet needs for patients with advanced cancer. Fatigue has been inadequately discussed and undertreated (31) due to lack of agreement on its measurement, inadequate understanding of the biology, and difficulty in conducting clinical trials of fatigue interventions (32). According to systematic meta-analyses and recently published studies, evidence-based management of cancer-related fatigue should be focused on behavioral and psychological interventions (33, 34), since pharmacological intervention has shown limited effect. Also, literature report on cancer-related fatigue seems to be more pronounced in women than in men, especially at the end of life (35).

*ESAS* was confirmed in our experience as one of the most valuable tools for detecting type and intensity of symptoms in metastatic cancer patients. In line with literature data (36), nearly one-fourth of the patients with advanced cancer in our cohort experienced three or more symptoms with an intensity of 7/10 or greater, exhibiting a strong correlation with survival. ESAS is confirmed as an important tool for identifying a group of patients with high symptom burden who require immediate support and assistance by the palliative care team (36, 37). Assessment of patient’s needs allows providing more effective support, relevant to every person’s individual experience, and it is necessary for setting priorities for resource allocation (31).

With regard to *SAT at the end of life*, our study showed that 43.8% of the patients received anticancer therapy during the last 60 days of life, of which 15% was within the last 30 days and 3.1% within the last 2 weeks of life. These figures can be partially justified by the good KPS that, on average, patients presented at the time of their first SCOC visit and that guides oncologists in their decision-making. In fact, almost half of the patients who received SAT in the last 30 days of life are therapy naïve. Furthermore, younger patients were more often treated with SAT at the end of life likely due to oncologists’ attitude of offering at least one opportunity for treatment even though cancer was in the advanced stage at diagnosis. SAT use in the last 14 days in our series compares favorably with the rate of 7% reported by Bakitas (4), 9.3% of an Italian study (38), as well as the rate of 13.6% observed by Greer et al. (39). Based on the analysis of SEER-Medicare, an overly aggressive care is associated with more than 10% of patients receiving SAT in the last 14 days (40). As for the patients who received SAT within 30 or 60 days, our results are in line with those reported in the literature in Italy (41, 42) and in other countries (43–45). Although the use of SAT in patients who are close to death has been increasing over time (46), little information is available about the clinical effect of such treatment (47). The extent of the contribution of SAT at the end of life and the role of advanced state of disease *per se* in hastening patients’ death cannot be further assessed in our experience, just like other reports in the literature, i.e., Zhu et al. (44). Indeed, our data confirm those by an American report in which SAT in the last 30 days of life was associated with an increased rate of death in the hospital (48, 49). Interestingly, older patients were found to receive less SAT during the last 60 days of life, in line with literature data (44, 48); they lived longer and died more frequently in hospice or at home. Indeed, as reported by Wright et al., perceptions of better end-of-life care are associated with earlier hospice enrolment, avoidance of ICU admission in the last 30 days of life, and deaths outside the hospital, among family members of elderly patients who died with lung or colorectal cancer (50). These findings are supportive of advance care planning consistent with patients’ preferences (50, 51) and may help both granting patients’ wishes regarding the place of death (52) as well as reducing caregivers’ distress (53).

The *role of oncologists* is strategic not only for the proper management of SCOC patients but also for an accurate estimation of prognosis in order to avoid therapeutic aggressiveness at the end of life when not justified. Continuing education of medical oncologists in palliative care remains critical for both providing the first level of palliative care, with systematic use of PROMs in clinical practice, and facilitating early access to an integrated SCOC (13). Prognosis, indeed, needs to be taken into account in the decision-making process, and several tools may help in the prognosis assessment, such as the Pap score (54). A systematic review of mortality predictors in patients with advanced cancer has been recently published (55). The “surprise question” and general clinical and laboratory variables are non-tumor-specific predictors of mortality within 3–24 months in patients with advanced cancer. This translates in the recommendation to pay more attention in the advanced stage of disease to clinical and laboratory parameters, which are not cancer-related, rather than to the type of tumor. In fact, a new validated machine-learning model to predict 6-month prognosis in patients with advanced solid tumors has been proposed (56), which can be useful and may support shared decision-making discussions between oncologists and patients with regard to considering a further line of SAT.

In addition, our data prove that there are *significant differences with regard to gender and age*. Women experienced a higher frequency of pain, anxiety, depression requiring psychological support in 22% of the subjects, loss of appetite, and higher MUST score values (100% requiring nutritional support), together with a higher frequency of total awareness of cancer prognosis compared with men. In particular, subgroup analysis by DT results showed that adult women had a significantly higher median distress compared with the rest of the cancer patient population.

Evaluation by age revealed a significantly lower median survival in younger subjects; in the same group, prevalence of pain was higher, along with awareness of prognosis. Such differences were confirmed in *multivariate analysis*. Indeed, age and gender might be differently impacted by early palliative care interventions, as reported in the study by Nipp et al. (57). No significant differences were found in our cohort by disease subgroups, except for lower dyspnea and lower survival in patients with GI cancer, as well higher pain and lower wellbeing in patients with GU cancer. This suggests, as reported by Chalkidis et al. and confirmed by a systematic review of mortality predictors in patients with advanced cancer, that patients with advanced solid tumors may converge to a common pathway at end of life, regardless of the cancer type, at which point patient-specific factors unrelated to cancer are the most important (55, 56).

In conclusion, to summarize it with the metaphor that Zimmermann has recently proposed, our study confirms the importance of introducing early palliative care as an umbrella for cancer patients and caregivers that must be opened before it starts to rain (58).

### Limitations

This study has some limitations. Although the results are in line with other similar reports in the literature, data collection was limited to one single center, which restricts the extrapolation of the results to the general population. Given the observational nature of this study, it was not possible to evaluate the effectiveness of this approach in comparison with a control group. The oncologist’s reasons for referring patients to SCOC and anticancer treatment decision-making were not factored in, although they would provide additional information.

### Implications

Our data confirm the importance of assessing PROMs by oncologists in clinical practice for a thorough evaluation of type and extent of needs of advanced cancer patients undergoing systemic cancer treatment. Oncologists must also be trained in the use of validated prognostic tools in order to refrain from proposing anticancer therapy at the end of life when not indicated. A fully embedded model in which the oncologist evaluates patients with advanced stage disease together with a palliative care team can facilitate the patients’ approach to palliative care and allows for direct sharing among the palliative care team regarding treatment options, life expectancy, and patient awareness of prognosis. Proper resources should be allocated in order to fulfill model requirements (59).

## Conclusions

Our data confirm the importance of assessing PROMs in order to acknowledge the type and extent of needs of advanced cancer patients. Italian cultural, social, and healthcare background may partly justify the low prevalence of social and spiritual issues detected in a relevant group of cancer patients with advanced stage of disease, and confirm the general presence of good family support, which is also assessed by the high percentage of patients who died at home (37.8%). Our study identified two subgroups of patients who require special attention and support due to the important symptom’s burden detected by PROMs: women who experienced higher frequency of pain, anxiety, depression, loss of appetite, and higher MUST scores, together with a higher frequency of total awareness of the prognosis; and younger adult subjects who have a shorter life expectancy, experience more intense pain and nausea, are more aware of the prognosis, and die more often in a hospital. These categories of patients with advanced stage of disease, regardless of the tumor type, require special attention to provide early referral to SCOC for adequate symptoms’ relief and proper care planning.

The overall SCOC performance was good as evaluated by some parameters such as the low percentage of patients receiving SAT at the end of life, the place of death, and the number of unplanned visits at the ER.

The role of the oncologist remains crucial to identify all patients in need of early palliative care through the systematic use of PROMs, which are now part of clinical practice (60). Assessment of patients’ needs should be done across the board on all patients with metastatic cancer, and then, through joint evaluation at SCOC, the categories of patients in greatest need can be identified. Changing perspective in the evaluation of patients is mandatory for oncologists in order to intercept the true needs of patients with cancer in advanced disease. Defining an accurate estimate of prognosis remains strategic in order to avoid SAT at the end of life, especially as second or further treatment line, which can contribute to being detrimental for patients’ survival and/or the quality of life for patients and caregivers.

## Data availability statement

The raw data supporting the conclusions of this article will be made available by the authors, without undue reservation.

## Ethics statement

This study was reviewed and approved by Veneto Institute of Oncology Ethics Committee. Written informed consent for participation was not required for this study in accordance with the national legislation and the institutional requirements.

## Author contributions

Conceptualization, VZ, AG, SS, AB, and SL. Data collection, VZ, AG, SS, MN, IG, AP, RM, MB, SM, CS, DM, FD, CDT, CP, BC, AAP, DB, FN, MC, FB, RI, AB and SL. Statistical analysis: CDT; original draft preparation, VZ, AG, AB, CDT, SS, and SL. Review and editing, all authors. All authors contributed to the article and approved the submitted version.

## Funding

This study was supported by Ricerca Corrente 2022, Italian Ministry of Health and by a charitable donation by Maria Sanvido.

## Acknowledgments

We thank Dr. Leonardo Trentin for essential contribution in setting up the Simultaneous Care Outpatient Clinic.

## Conflict of interest

The authors declare that the research was conducted in the absence of any commercial or financial relationships that could be construed as a potential conflict of interest.

## Publisher’s note

All claims expressed in this article are solely those of the authors and do not necessarily represent those of their affiliated organizations, or those of the publisher, the editors and the reviewers. Any product that may be evaluated in this article, or claim that may be made by its manufacturer, is not guaranteed or endorsed by the publisher.
